# Modulation of Microenvironment Signals by Proteolytic Shedding of Cell Surface Extracellular Matrix Receptors

**DOI:** 10.3389/fcell.2021.736735

**Published:** 2021-11-02

**Authors:** Yoshifumi Itoh

**Affiliations:** Kennedy Institute of Rheumatology, University of Oxford, Oxford, United Kingdom

**Keywords:** integrin, syndecan, CD44, DDR1, shedding

## Abstract

Multicellular organisms are composed of cells and extracellular matrix (ECM). ECM is a network of multidomain macromolecules that fills gaps between cells. It acts as a glue to connect cells, provides scaffolding for migrating cells, and pools cytokines and growth factors. ECM also directly sends signals to the cells through ECM receptors, providing survival signals and migration cues. Altogether, ECM provides a correct microenvironment for the cells to function in the tissue. Although ECM acts as a signaling molecule, they are insoluble solid molecules, unlike soluble receptor ligands such as cytokines and growth factors. Upon cell binding to the ECM through ECM receptors and signals transmitted, cells then need to have a mechanism to release from ECM to prevent prolonged signals, which may be tumorigenic, and migrate on ECM. One effective means to release the cells from ECM is to cleave the ECM receptors by proteinases. In this mini-review, current knowledge of ECM receptor shedding will be discussed.

## Introduction

**FIGURE 1:**
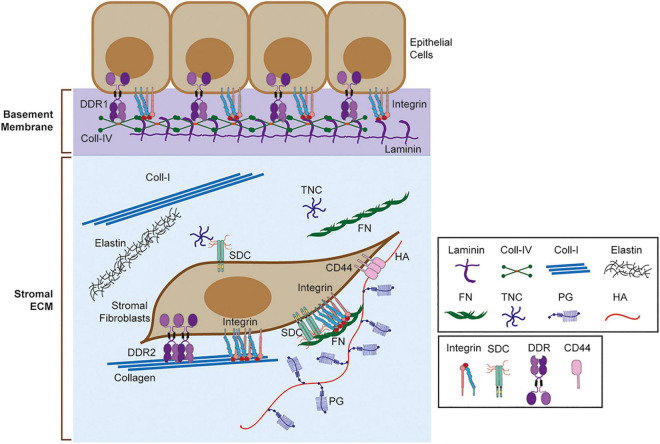
Extracellular matrix (ECM) and ECM receptors. Schematic representation of basement membrane and stromal ECM and ECM receptors. Selected ECM molecules are shown here including laminin, collagen IV (Coll-IV), collagen I (Coll-I), elastin, fibronectin (FN), tenascin C (TNC), proteoglycan (PG), and hyaluronic acid (HA). ECM receptors, including integrin, sydecans (SDC), discoidin domain receptors (DDRs), and CD44 are also shown. Integrins bind different matrix, including collagen, laminin, and FN. SDC binds TNC and FN through heparan sulfate (HS) chains. DDR1 binds to fibrillar collagens (Coll-I, Coll-II, Coll-III) and Coll-IV, while DDR2 binds to fibrillar collagens only. CD44 binds to HA.

Multicellular organisms consist of cells and extracellular matrix (ECM). ECM is a network of multidomain macromolecules, and the composition, biological and mechanical properties of ECM are different in each organ and tissue, providing appropriate functionalities (Theocharis et al., 2019). ECM acts as a glue to connect cells, fills the gaps between cells, provides structural support for the organs, divides tissue compartments, acts as a scaffolding for migrating cells, pools growth factors and cytokines, and provides signals directly to the cells (Theocharis et al., 2019; Piperigkou et al., 2021). Cells sense surrounding ECM and modify it by degrading them, synthesizing them, and depositing them. Altogether, ECM provides a correct microenvironment for the cells to function in the tissue (Theocharis et al., 2019; Piperigkou et al., 2021). Components of ECM include collagens, fibronectin, laminins, elastin, tenascin, proteoglycans, and glycosaminoglycans (Figure 1; Karamanos et al., 2019; Theocharis et al., 2019). Function, property, and the tissue that express these components are described in detail in the recent review articles (Karamanos et al., 2019; Theocharis et al., 2019). Each organ and tissue provide a unique composition of ECM, creating a proper microenvironment for the cells and organs to function.

Cells recognize ECM by ECM receptors, which can also act as adhesion molecules (Theocharis et al., 2019). Initiating signaling by binding to the ECM is important, but it is equally important to terminate signals, especially for receptor tyrosine kinases. Aberrantly prolonged signals may cause tumorigenesis as seen by the oncogene. Since ECM receptors are also cell adhesion molecule, they also need to be dissociated from ECM to migrate in the ECM. Thus, disengaging is an another crucial step for the regulation of both cell signaling and cell migration (Gifford and Itoh, 2019). One mechanism is the proteolytic cleavage of the ECM receptor to dissociate cell-ECM interaction. Many examples have been reported to date, and it has been shown that this is a crucial step in the regulation of ECM receptors. In this mini-review, current knowledge of proteinase-mediated cell-ECM modulation is discussed as it is one of the dynamic regulatory mechanisms of ECM signaling and cell adhesion.

## Integrins

Integrins are a hetero-dimeric non-covalent complex of α and β subunits. There are 18 types of α subunits and 8 types of β subunits, and with the different combinations, there are at least 24 unique integrins (Bouvard et al., 2013; Kechagia et al., 2019). Integrins are important cell adhesion molecules, supporting cell migration, and major ECM receptors that transmit biomechanical signals from the microenvironment (Bouvard et al., 2013; Kechagia et al., 2019).

**FIGURE 2:**
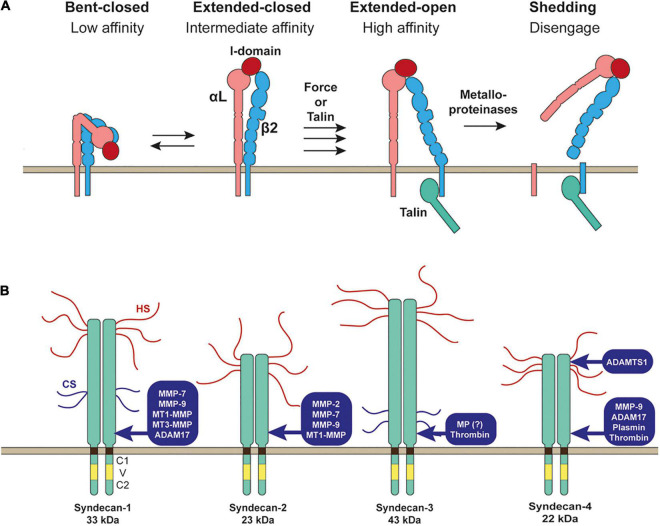
Shedding of LFA-1 (αLβ2 integrin) and syndecans. **(A)** Inactive bent-closed conformation of LFA-1 gets activated to extended-open conformation through external force or binding of talin at the cytoplasmic domain of β2 integrin. LFA-1 is then cleaved by metalloporteinase(s) to release both αL and β2 subunits to disengage LFA-1-mediated cell adhesion. **(B)** Proteinases reported to shed syndecans are depicted.

Integrins expressed on the cell surface need to be activated by changing the conformation from the bending inactive conformation to stretched active conformation to bind ECM stably (Figure 2A). There are two different signaling pathways that activate the integrins: inside-out and outside-in signals (Bouvard et al., 2013; Kechagia et al., 2019). Inside-out signals involve the binding intracellular adaptor proteins to the cytoplasmic tail of the integrin β-subunit, such as talin (Bouvard et al., 2013; Kechagia et al., 2019). This binding event induces separation of the cytoplasmic domains of integrin α and β subunits and triggers a global conformational change of the extracellular domain from the bending to stretched conformation (Figure 2A; Bouvard et al., 2013; Kechagia et al., 2019). Outside-in signals involve the initial weak interaction of inactive integrin with ECM followed by the application of external force by cell movement or shear stress, inducing a conformational change of the integrin to a stretched active conformation (Bouvard et al., 2013; Kechagia et al., 2019; Figure 2A).

Recognition of ECM by integrin is fundamental for cell migration and mechanical force transmission, but dissociating integrins from the ECM is also crucial for cell migration. Dissociation of integrins from ECM can also be regulated by inside-out signaling by converting the integrin conformation to an inactive form. This inactivation process of the integrin involves binding of ICAP1 (integrin cytoplasmic domain-associated protein-1) or filamin at the cytoplasmic tail of integrin β-subunit that compete out the talin binding or binding of SHARPIN or MDGI (mammary-derived growth inhibitor) at the cytoplasmic tail of integrin α-subunit (Bouvard et al., 2013; Kechagia et al., 2019).

Integrin disengagement does not seem to require proteolytic shedding of integrins in general as described above. However, there are several reports showing integrin shedding. It has been reported that lymphocyte function-associated antigen-1 (LFA-1, αLβ2, CD11a/CD18) is shed as a heterodimer from the cell surface of leukocytes and monocytes upon inflammatory response (Evans et al., 2006). αMβ2 (MAC-1, CD11b/CD18) was also shown to be shed by a metalloproteinase (Gomez et al., 2012). It was previously reported that matrix metalloproteinase 9 (MMP-9) was responsible for β2 integrin shedding in macrophages (Vaisar et al., 2009). However, αMβ2 shedding still occurred in MMP-9 null, a disintegrin and metalloproteinase (ADAM)10 null or ADAM17 null mice, suggesting that the responsible sheddase of αMβ2 is other metalloproteinases (Gomez et al., 2012). It was proposed that LFA-1 shedding may play a role in leukocyte detachment after trans-endothelial migration, regulating integrin-dependent outside-in signaling (Gomez et al., 2012). β1 integrin was also shown to be shed by a metalloproteinase in microvessel endothelial cells upon treatment of the cells with homocysteine, a risk factor of cardiovascular diseases (Shastry and Tyagi, 2004). It was found that the shedding is inhibited by tissue inhibitor of metalloproteinases 4 (TIMP-4), which inhibits all MMPs and some ADAMs, indicating that responsible metalloproteinase is a TIMP-4-sensitive metalloproteinase (Shastry and Tyagi, 2004). However, the biological significance of β1 integrin shedding is not depicted. Further investigation on the integrin shedding is needed to reveal its significance.

## Syndecans

Syndecans are type 1 transmembrane heparan sulfate proteoglycans (HSPGs). The heparan sulfate (HS) chains at the extracellular domains interact with different ligands, including ECM glycoproteins, cytokines, chemokines, and growth factors (Couchman et al., 2015; Gharbaran, 2016; Afratis et al., 2017; Gondelaud and Ricard-Blum, 2019). There are four syndecans 1–4. Syndecan-1 is highly expressed in epithelia, syndecan-2 in endothelia and fibroblasts, syndecan-3 is mainly expressed in neuronal tissue and some musculoskeletal tissue, while syndecan-4 can be found in most tissue. A single cell can express multiple syndecans. Syndecan core proteins are 33 kDa for syndecan-1, 23 kDa for syndecan-2, 43 kDa for syndecan-3, and 22 kDa for syndecan-4. Each syndecan is attached by three HS chains, and syndecan-1 and syndecan-3 are attached by other two chondroitin sulfate chains (Figure 2B; Couchman et al., 2015; Gharbaran, 2016; Afratis et al., 2017; Gondelaud and Ricard-Blum, 2019). The transmembrane domain of all syndecans contains a GXXXG motif that promotes the formation of SDS (sodium dodecyl sulfate)-resistant homo-dimers (Choi et al., 2005; Dews and Mackenzie, 2007), and this transmembrane domain-mediated homo-dimerization has been reported to be crucial for the function of syndecan-2 and syndecan-4 (Choi et al., 2005).

Syndecans are known to be shed by many different proteinases (Manon-Jensen et al., 2010). Syndecan-1 was shown to be shed by MMP-7 (Chen et al., 2009), MMP-9 (Brule et al., 2006), membrane-type 1 MMP (MT1-MMP), MT3-MMP (Endo et al., 2003), and ADAM17 (Pruessmeyer et al., 2010). Syndecan-2 was shown to be shed by MMP-2, MMP-9 (Fears et al., 2006), MMP-7 (Choi et al., 2012), and MT1-MMP (Lee et al., 2017). Syndecan-3 sheddase was a metalloproteinase, but it has not been identified yet (Asundi et al., 2003). Serine proteinase, thrombin, was also shown to cleave syndecan-3 (Jannaway et al., 2019). Syndecan-4 is shed by MMP-9 (Brule et al., 2006), ADAM17 (Pruessmeyer et al., 2010), ADAMTS1 (Rodriguez-Manzaneque et al., 2009), plasmin (Schmidt et al., 2005), and thrombin (Schmidt et al., 2005; Jannaway et al., 2019).

Syndecan shedding has two biological effects. First, it decreases syndecan levels on the cell surface. Several growth factors are known to interact with HS chain of the syndecans, including fibroblasts growth factor (FGF), vascular endothelial growth factor (VEGF), epidermal growth factor (EGF), hepatocyte growth factor (HGF), platelet-derived growth factor (PDGF), and transforming growth factor β1 (TGFβ1), Wnt, and Hedgehog (Hh) (Chung et al., 2016). This interaction is essential for their signaling to present them to their receptors. It has been shown that HS-bound FGF2 increased the affinity for FGFR by over one magnitude: *K*_*d*_ of 41 nM went to 4.9 nM (Pantoliano et al., 1994). Thus, loss of syndecan by shedding would greatly influence the presentation of growth factors to the receptors. The second effect is that the shed ectodomain of syndecans can act as a soluble factor that exerts biological function. For instance, shed soluble syndecan-1 from fibroblasts can mediate mitogenic responses in human breast cancer cells. This paracrine event is mediated by HS chain, FGF2, and stromal-derived factor 1 (SDF1) (Su et al., 2007). Co-culture of fibroblasts and T47D breast cancer cells resulted in T47D proliferation. It was found that shed syndecan-1 from fibroblasts was necessary to promote SDF1 and FGF2 dependent T47D growth (Su et al., 2007). Another example can be that shed syndecan-2 deposited to ECM can be a ligand for the protein tyrosine phosphatase receptor CD148 to promote β1 integrin-mediated cell adhesion (Whiteford et al., 2011). Syndecan 1 and 4 were shown to modulate Wnt signaling by binding Wnt at the HS chain for development, stem cell differentiation, and cancer progression (Chung et al., 2016; Onyeisi et al., 2021). Another example was reported on thrombin-mediated syndecan-3 and 4 shedding. In endothelial cells, Syndecan-3 and syndecan-4 were shown to be shed by thrombin, and shed fragments bound to the cells and caused VE-cadherin disorganization, causing paracellular hyperpermeability (Jannaway et al., 2019). Thus, shedding of the syndecans impacts different biological events significantly.

## CD44

CD44 is a type I transmembrane cell adhesion molecule whose ligand is hyaluronic acid (HA), a glycosaminoglycan (Naor et al., 1997; Cichy and Pure, 2003; Ponta et al., 2003). CD44 was also shown to bind osteopontin (Weber et al., 1996), fibronectin, type I collagen (Jalkanen and Jalkanen, 1992), type IV collagen (Knutson et al., 1996), and matrix metalloproteinases (MMPs) (Yu and Stamenkovic, 1999). CD44 is expressed in most of the cell types in our body, and a shed form of soluble CD44 has been detected in circulation and other body fluids (Ponta et al., 2003). CD44 is encoded by a single gene, but multiple isoforms are generated by alternative splicing. CD44 gene contains 20 exons, and the most common form of CD44 referred to as standard or hematopoietic CD44 and is encoded by 10 exons (Naor et al., 1997; Ponta et al., 2003; Chen et al., 2018). This form is the shortest isoform. Other forms have insertion of alternative exons (V2-V10) at a single site within the membrane proximal region of the ectodomain (Naor et al., 1997; Ponta et al., 2003; Chen et al., 2018). Interestingly, CD44 with V3 insertion made CD44 modified with HS, which may provide additional functionality to the receptor: HB-EGF presentation (Bennett et al., 1995).

CD44 consists of N-terminal HA-binding globular domain, followed by a stem that has glycosylation and GAG binding sites, transmembrane domain, and the cytoplasmic tail, which binds to band 3.1 proteins (ERM proteins) linking CD44 to actin cytoskeleton (Ponta et al., 2003; Figure 2A). It has been reported that CD44 can undergo transition to a high affinity state upon stimulation of the cells by soluble factors, such as TNFα (tumor necrosis factor α), Oncostatin M, TGFβ, and IFNγ (interferon γ) (Levesque and Haynes, 1997; Cichy and Pure, 2000; Brown et al., 2001). Since TNFα-induced CD44 activation was inhibited by NaClO3, a sulfation inhibitor that blocks the transfer of sulfate to 3’-phosphoadenosine 5’-phosphosulfate, a universal sulfate donor (Brown et al., 2001), sulfation of CD44 may play a role. However, detailed molecular events on CD44 molecule during the activation process are not fully understood.

**FIGURE 3:**
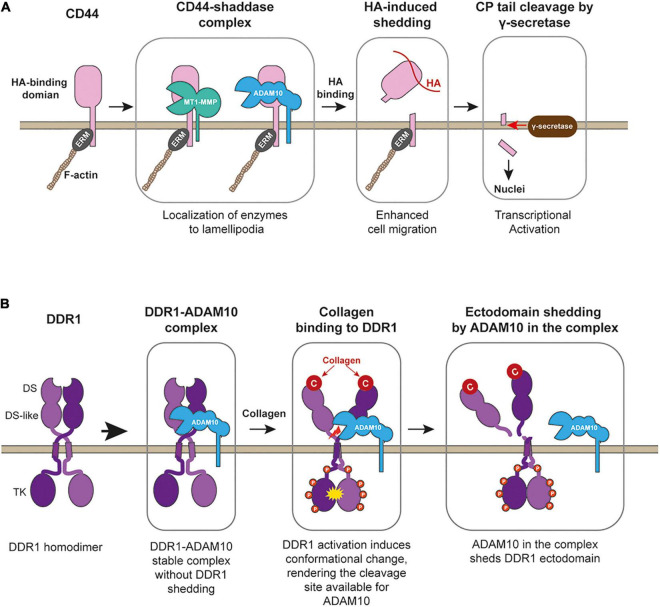
CD44 processing and collagen-induced shedding of DDR1 by ADAM10. **(A)** CD44 forms stable complex with MT1-MMP (or potentially with ADAM10), allowing the enzymes to localize at lamellipodia. Upon binding to HA-substratum at the leading edge, the enzyme in the complex shed the ectodomain of CD44, releasing CD44-mediated cell adhesion and promote cell migration. After ectodomain removal, γ-secretase releases the cytoplamic tail of the CD44, and released cytoplasmic tail acts as a transcriptional factor to stimulate gene expression. ERM, band 3.1 proteins. **(B)** DDR1 upon secretion forms a stable complex with ADAM10, but ADAM10 does not cleave DDR1. Collagen binding activates DDR1 and induces conformational change in its ectodomain, rendering the cleavage site available to ADAM10. ADAM10 cleaves between Pro407-Val408 at the juxtamembrane region to release DDR1-dependent cell adhesion and to stop collagen signaling through DDR1.

CD44 was shown to play a role in inflammation, as administration of anti-CD44 antibody protected mice from experimental arthritis (Mikecz et al., 1995). It has been also shown that CD44 plays a crucial role in cancer progression (Chen et al., 2018). In both inflammatory disease and cancer patients, proteolytically generated soluble CD44 levels in the circulation are much higher. Thus, CD44 shedding has been suggested to link to disease progressions. There are at least three CD44 sheddases identified, MT1-MMP (MMP-14) (Kajita et al., 2001), ADAM10, and ADAM17 (Okamoto et al., 1999). MT1-MMP-dependent shedding occurs constitutively at the lamellipodia when CD44 and MT1-MMP are co-expressed in the cells (Kajita et al., 2001). It was found that CD44 shedding by MT1-MMP promoted cancer cell migration on HA-based substratum (Kajita et al., 2001). CD44 interacts with MT1-MMP through its stem region and the hemopexin (Hpx) domain of MT1-MMP (Mori et al., 2002). It has been shown that CD44 localizes at the lamellipodia regulated through Rho GTPAses (Okamoto et al., 1999). Thus, interaction with CD44 allows MT1-MMP to localize at the lamellipodia (Mori et al., 2002). ADAM10- and ADAM17-dependent CD44 shedding was shown to be induced by Calcium influx or protein kinase C activation, respectively (Okamoto et al., 1999; Nagano et al., 2004). However, when cell migration on HA-based matrix was measured, knockdown of ADAM10 or 17 in human lung adenocarcinoma inhibited the migration by 75% in both (Nagano et al., 2004), suggesting that ADAM-dependent CD44 shedding also supports cell migration on HA matrix. Since CD44 is localized at the lamellipodia, and suppression of Rac1 by overexpressing Rac1 dominant-negative mutant inhibited the shedding (Okamoto et al., 1999), CD44 shedding by these proteinases also occurs at the lamellipodia. It has been reported that the addition of HA to the cells initiated CD44 shedding (Sugahara et al., 2003), suggesting that CD44 shedding may occur at the leading edge where CD44 binds to HA-containing substratum (Figure 3A). In supporting this, it was shown that ADAM10 and CD44 were found to be colocalized at the ruffling membrane structures in human melanoma cells (Anderegg et al., 2009).

It was shown that after shedding of the ectodomain by a metalloproteinase, the soluble intracellular domain of CD44 was released by presenilin-dependent gamma-secretase and act as transcriptional activator (Figure 3A; Murakami et al., 2003; Pelletier et al., 2006). This intracellular fragment was also shown to induce oncogenic transformation of rat fibroblasts, which was mediated by a receptor tyrosine kinase, Ret. However, the mechanism of the fragment-induced Ret activation was not understood (Pelletier et al., 2006). Thus, CD44 ectodomain shedding triggers transformation as well. Taking together, the membrane proteinase-dependent shedding is likely the core of CD44-mediated cell migration. As described above, CD44 is cleaved by three membrane-bound metalloproteinases, and all of these shedding promotes cell migration. However, it is not clear which shedding events plays a role in different types of cell migration. In human melanoma cells, constitutive shedding of CD44 was reported to be mediated by ADAM10 but not by MT1-MMP or ADAM17 despite all of these enzymes were expressed in the cells (Anderegg et al., 2009). Further clarifications are required in the future.

## Discoidin Domain Receptors

Discoidin domain receptors (DDRs) are collagen receptor tyrosine kinases, and there are two types, DDR1 and DDR2. Both DDRs have collagen-binding discoidin domain at the N-terminus of the ectodomain and tyrosine kinase domain at their cytoplasmic domain (Leitinger, 2014). DDRs are the only receptor tyrosine kinase whose ligands are collagens. Both DDRs bind to fibrillar collagens, but DDR1 additionally binds to type IV collagen (Leitinger, 2014). Under physiological condition, DDR1 is expressed in epithelial cells, while DDR2 is expressed in mesenchymal cells. DDR1 has five splice variants in which DDR1a, DDR1b, and DDR1c are functional DDR1s, while DDR1d and DDR1e are non-functional receptors lacking tyrosine kinase domain. DDR1a (867 AA) is the shortest of the three functional DDR1s, and DDR1b (913 AA) has 37 amino acid insertion in the juxtamembrane region in the cytoplasmic domain and is the predominant isoform expressed during embryogenesis. DDR1a shows higher expression in several human mammary cancer cells. DDR1c (919 AA) is the longest isoform with six additional amino acids insertion in the kinase domain relative to DDR1b. DDR2 has only one gene product.

DDR1 and DDR2 bind to the “GVMGFO” motif (Konitsiotis et al., 2008; Xu et al., 2011) within collagens I, II, and III, which is distinct from β1 integrin binding site “GFOGER” (Emsley et al., 2000). Thus, the binding of DDRs and integrins are independent. For DDRs to bind collagens, they need to form a homodimer (Leitinger, 2003). DDR1 dimerization is likely to be mediated through the leucine zipper in the transmembrane domain (Noordeen et al., 2006), while DDR2 ectodomain spontaneously forms a dimer (Leitinger, 2003). Thus, ligand binding-induced dimerization, which is found in many receptor tyrosine kinases, does not apply to DDRs. It has been shown that further clustering of dimer DDRs occurs upon collagen binding. Interestingly, inter-DDR dimer phosphorylation was shown to occur between DDR1s and between DDR1 and DDR2 (Juskaite et al., 2017).

It was shown that the DDR1 ectodomain is proteolytically shed upon collagen treatment of the cells, which can be inhibited by broad-spectrum metalloproteinase inhibitor (Vogel, 2002). Later, the responsible enzyme was identified as type-I transmembrane proteinse, ADAM10 (Shitomi et al., 2015). Interestingly ADAM10 and DDR1 exist as a stable complex in the *cis* arrangement on the cell surface, but the shedding does not occur unless collagen binds to DDR1 (Figure 3B). Since the interaction of DDR1 with collagen cannot be controlled by inside-out signaling like integrins, the ectodomain shedding is the only means to cancel the DDR1-mediated collagen adhesion. It was shown that shedding-deficient DDR1 had a much longer half-life of collagen-induced tyrosine phosphorylation (Shitomi et al., 2015), suggesting that DDR1 shedding controls the duration of collagen signaling. DDR1-mediated collagen signal has been shown to increase cell motility (Lu et al., 2011). When cells migrate on the collagen matrix, adhesion to the collagen matrix is essential, but dissociation from the collagen is equally essential, and ADAM10-dependent DDR1 shedding plays a key role. Inhibition of DDR1 shedding by ADAM10 significantly inhibited epithelial cell migration on the collagen matrix (Shitomi et al., 2015). Besides ADAM10, MT1-MMP was also reported to shed a DDR1 ectodomain. It was shown that co-expression of MT1-MMP with DDR1 in COS7 cells caused constitutive shedding of DDR1 ectodomain (Fu et al., 2013). However, this event was not shown in endogenous MT1-MMP and DDR1 (Fu et al., 2013). Thus, further investigation is necessary to examine the role of MT1-MMP in DDR1 shedding.

DDR2 must also be dissociated from collagen upon transmitting collagen signals, but so far, DDR2 shedding has not been described, and an alternative mechanism has not been identified.

## Concluding Remarks

Proteolysis is an irreversible reaction, which is different from other signal transduction events such as phosphorylation. Once a molecule is cleaved, the reaction cannot be reversed for this cleaved molecule. However, membrane proteins, including ECM receptors, can be replenished by newly synthesized or recycled molecules on the cell surface. Therefore, proteolytic shedding of ECM receptors can effectively and dynamically regulate cell signaling and cell adhesion.

For membrane proteins to be shed by proteinases, their interaction is crucial for presenting the cleavage sites to the enzymes. For instance, MT1-MMP forms a complex with CD44 through the hemopexin domain (Mori et al., 2002), and ADAM10 was also shown to form a complex with DDR1 (Shitomi et al., 2015). Interestingly, these proteinase-ECM receptor complexes are found to be stable, and these enzymes in the complex do not immediately shed their interaction partners as described above. The shedding event can be triggered by the interaction of the ECM receptor with the ligand ECM. A good example is DDR1-shedding by ADAM10. ADAM10 and DDR1 are found to be in a stable complex when expressed on the cell surface, and the DDR1 shedding only takes place upon DDR1 binds to collagen (Shitomi et al., 2015). Such complex formation of sheddases and ECM receptors and ligand-induced ECM receptor shedding are essential for dynamic regulation of microenvironment signal and cell migration.

Besides ECM receptor shedding, ECM degradation by proteinases also affects cell adhesion properties. For instance, the formation of focal adhesion on gelatin substratum was shown to be disturbed by MT1-MMP expression. MT1-MMP localizes at focal adhesion and degrades gelatin, destabilizing integrin-dependent focal adhesion (Takino et al., 2006; Woskowicz et al., 2013). It is possible that such activity may contribute to disengagement process of integrin-dependent cell adhesion and cell migration.

Nevertheless, proteinase-mediated shedding of ECM receptors plays a significant role in regulating microenvironment ECM signaling and cell migration. However, detailed molecular mechanisms of shedding events and the biological significance of each shedding in health and diseases still need to be investigated. There are some controversies as well. Further understanding of each process of proteolytic shedding of ECM receptors may reveal novel concepts and identify the target process for the disease treatment in the future.

## Author Contributions

YI wrote the manuscript.

## Conflict of Interest

The author declares that the research was conducted in the absence of any commercial or financial relationships that could be construed as a potential conflict of interest.

## Publisher’s Note

All claims expressed in this article are solely those of the authors and do not necessarily represent those of their affiliated organizations, or those of the publisher, the editors and the reviewers. Any product that may be evaluated in this article, or claim that may be made by its manufacturer, is not guaranteed or endorsed by the publisher.
